# Divergent effects of sex and calcium/vitamin D supplementation on serum magnesium and markers of bone structure and function during initial military training

**DOI:** 10.1017/S0007114521004669

**Published:** 2022-11-14

**Authors:** Stephen R. Hennigar, Alyssa M. Kelley, Anna T. Nakayama, Bradley J. Anderson, James P. McClung, Erin Gaffney-Stomberg

**Affiliations:** 1 Florida State University, Department of Nutrition and Integrative Physiology, Tallahassee, FL, USA; 2 US Army Research Institute of Environmental Medicine (USARIEM), Military Nutrition Division, Natick, MA, USA; 3 Oak Ridge Institute for Science and Education, Belcamp, MD, USA; 4 USARIEM, Military Performance Division, Natick, MA, USA

**Keywords:** Serum magnesium, Magnesium status, Reference interval, Dietary reference intakes

## Abstract

Maintaining Mg status may be important for military recruits, a population that experiences high rates of stress fracture during initial military training (IMT). The objectives of this secondary analysis were to (1) compare dietary Mg intake and serum Mg in female and male recruits pre- and post-IMT, (2) determine whether serum Mg was related to parameters of bone health pre-IMT, and (3) whether Ca and vitamin D supplementation (Ca/vitamin D) during IMT modified serum Mg. Females (*n* 62) and males (*n* 51) consumed 2000 mg of Ca and 25 μg of vitamin D/d or placebo during IMT (12 weeks). Dietary Mg intakes were estimated using FFQ, serum Mg was assessed and peripheral quantitative computed tomography was performed on the tibia. Dietary Mg intakes for females and males pre-IMT were below the estimated average requirement and did not change with training. Serum Mg increased during IMT in females (0·06 ± 0·08 mmol/l) compared with males (–0·02 ± 0·10 mmol/l; *P* < 0·001) and in those consuming Ca/vitamin D (0·05 ± 0·09 mmol/l) compared with placebo (0·001 ± 0·11 mmol/l; *P* = 0·015). In females, serum Mg was associated with total bone mineral content (BMC, *β* = 0·367, *P* = 0·004) and robustness (*β* = 0·393, *P* = 0·006) at the distal 4 % site, stress–strain index of the polaris axis (*β* = 0·334, *P* = 0·009) and robustness (*β* = 0·420, *P* = 0·004) at the 14 % diaphyseal site, and BMC (*β* = 0·309, *P* = 0·009) and stress–strain index of the polaris axis (*β* = 0·314, *P* = 0·006) at the 66 % diaphyseal site pre-IMT. No significant relationships between serum Mg and bone measures were observed in males. Findings suggest that serum Mg may be modulated by Ca/vitamin D intake and may impact tibial bone health during training in female military recruits.

Mg is an essential mineral that imparts critical structural and functional roles in the body. Approximately 50–60 % of total body Mg is found in bone^(1)^ either conjugated to hydroxyapatite crystals or on the bone surface and is involved in bone cell function and hydroxyapatite crystal formation. Mg on the bone surface is thought to be an exchangeable Mg pool to help maintain serum Mg concentrations^(2)^. A small amount of Mg (1 %) is found extracellularly and the remaining is primarily found in soft tissue, mainly skeletal muscle^(1)^. Mg in this capacity is bound to phospholipids of cell membranes or associated with enzymes where it assists with membrane stabilisation or as a cofactor for enzymatic reactions, respectively.

In 2015, the Dietary Guidelines Advisory Committee found that approximately half of all Americans, and as many as 87 % of females aged 14–18 years were below the Estimated Average Requirement (EAR) for Mg (300 mg/d) and characterised Mg as a shortfall nutrient^(3)^. Currently, serum Mg concentration is used to assess Mg status of an individual with a reference interval of 0·75–0·95 mmol/l^(4)^; however, there is debate as to whether to increase the lower cut-off to 0·85 mmol/l^(5)^. Serum Mg concentrations within the current clinical cut-off for Mg deficiency (i.e. subclinical or chronic latent Mg deficiency) have been related to increased risk for many chronic diseases, including osteoporosis^(5,6)^. Epidemiological studies have reported a positive association between dietary Mg intake and bone mineral density^(7,8)^, and studies in rodents have documented direct effects of even marginal Mg restriction on bone health^(9–13)^. A meta-analysis found that low serum Mg concentrations were a risk factor for osteoporosis in postmenopausal women^(1)^.

Skeletal stress fractures are common during initial military training (IMT) but disproportionately affect female (8–21 %) as compared with male (2–5 %) recruits^(14)^. In fact, female sex is the most commonly identified risk factor for stress fracture^(14)^. Stress fractures are the result of repetitive mechanical deformation of the bone resulting in fatigue damage. During IMT, recruits experience unaccustomed, repetitive physical activity such as standing in formation, marching while carrying loads and running or sprinting. Mg requirements increase in individuals who routinely engage in strenuous physical activity, as Mg is involved in processes that affect muscle function such as energy production, nucleic acid and protein synthesis, muscle contraction, and oxygen uptake^(15,16)^. Moreover, physical activity may result in increased Mg excretion through sweat and urine^(16)^. Coupled with low dietary Mg intakes, these data indicate that military recruits may be vulnerable to chronic latent Mg deficiency and its associated symptoms, particularly detriments in bone health.

The primary objectives of this retrospective analysis of a randomised, double-blind, placebo-controlled trial were to (1) characterise dietary Mg intake and serum Mg concentrations in female and male recruits pre- and post-IMT and (2) determine whether serum Mg was associated with parameters of bone health in female and male recruits starting military training. The current analysis was conducted using a subset of participants enrolled in the previously published study which examined the effects of dietary Ca/vitamin D intake on bone health during military training^(17)^. Previous studies have demonstrated direct and indirect effects of vitamin D on intestinal Mg absorption^(18)^. Thus, a secondary objective was to determine whether Ca and vitamin D supplementation (Ca/vitamin D) during training modified serum Mg.

## Methods

### Participants

This was a secondary analysis of a study that examined the effects of Ca/vitamin D supplemention on bone health in recruits undergoing United States Marine Corps IMT^(17)^. The study conformed to the principles of the Declaration of Helsinki and was approved by the Institutional Review Board at the US Army Research Institute of Environmental Medicine and registered at www.clinicaltrials.gov as NCT02636348. A total of 113 participants (females, *n* 62; males, *n* 51) who completed Marine Corps Basic Combat Training at Parris Island, SC (32°N latitude), had serum available, and reported plausible energy intake in their FFQ (see dietary intake below) were used for this analysis.

### Intervention

Participants were block randomised by race and sex to one of two intervention groups: placebo or 2000 mg of Ca and 25 μg of vitamin D per d. These concentrations of Ca and vitamin D have been demonstrated to reduce stress fracture incidence in female Navy recruits provided capsules daily throughout training^(19)^. The current study expanded upon earlier findings by including both males and females undergoing training during two different seasons and evaluated effects of supplementation on circulating measures of bone health as well as imaging. Participants were assigned to receive food bars fortified with or without Ca/vitamin D. Ca was added to the bars in the form of calcium carbonate and vitamin D was added as D_3_. A composite of five placebo and Ca/vitamin D bars were sent to Covance Laboratories (Madison, WI) for Ca, vitamin D and Mg analysis by ICP Emission Spectrometry. Placebo bars contained 20 mg of Ca, <0·02 µg of vitamin D, and 23 mg of Mg and Ca/vitamin D bars contained 1032 mg of Ca, 13·7 µg of vitamin D, and 26 mg of Mg. The placebo and Ca/vitamin D bars were identical in taste and appearance and conformed to all ration standards for safety and stability. Bars were consumed between meals (two times/d). Compliance was 98·3 ± 2·5 % in the placebo group and 95·7 ± 8·5 % in the Ca/vitamin D group.

### Military training

Marine Corps Basic Combat Training consists of 12 weeks of physical and military-specific training. Physical requirements include aerobic activity such as foot marching with weighted packs, obstacle courses, distance running, and sprinting, and muscle strength training and callisthenic exercises. Recruits consume three self-selected meals/d in a dining facility during training and are not permitted to consume snacks (aside from the study intervention) or dietary supplements.

### Anthropometrics

All anthropometric measures were determined pre- and post-IMT, with the exception of height, which was measured at baseline to the nearest 0·1 cm using a stadiometer (Creative Health Products). Weight was determined to the nearest 0·1 kg on a calibrated digital scale (Belfour Scales) and BMI was calculated as body weight (kg)/height (m)^2^. Skin fold thickness was measured at the tricep, suprailiac, and abdomen for women and tricep, suscapula, and chest for men. Measurements were made in duplicate to the nearest mm. A third measurement was taken if the measurements differed by more than 2 mm. Body fat percentage was estimated using sex-specific three-site skinfold Jackson–Pollock equations^(20,21)^.

### Dietary intake

Pre- and post-IMT dietary intakes were estimated using a self-administered validated FFQ (Block 2014 FFQ; NutritionQuest) under the supervision of Registered Dietitians. Pre-IMT questionnaires were answered relative to how the recruits ate for the 6 months prior to IMT, and post-IMT questionnaires were answered relative to how the recruits ate during the 12 weeks of IMT as previously reported^(22)^. Nutrient intake data were excluded from the analysis if energy intake was implausible (<300 or >4500 kcals for females; <800 or > 5000 kcal for males)^(23)^.

### Blood collection and circulating biomarkers

Fasting blood samples were collected by antecubital venepuncture into vacuum tubes (Vacutainer; Becton Dickinson). Serum Mg was measured using a colorimetric kit (BioVision, Inc.). Intact parathyroid hormone (PTH) was measured by immunoassay (Siemens Immulite 2000). Ionised Ca (iCa) was measured using a handheld iSTAT^®^ System point-of-care device and Chem8 + Cartridges (Abbott Laboratories). 25-hydroxyvitamin D and 1,25-dihydroxyvitamin D were determined by RIA (DiaSorin Inc.). Procollagen type 1 N-terminal propeptide (P1NP, MyBioSource), c-telopeptide cross-links of type 1 collagen (CTX, Immunodiagnostic Systems), tartrate-resistant acid phosphatase 5b (TRAP5b, Quidel) and bone-specific alkaline phosphatase (BAP, Quidel) were analysed by ELISA.

### Peripheral quantitative computed tomography

Pre- and post-IMT peripheral quantitative computed tomography scans of the tibia were performed using the Stratec XCT-2000 or −3000 (Stratec Medizintechnik GmbH) as described previously^(24)^. Briefly, tibia length was measured on the non-dominant leg from the bottom of the medial malleolus to the tibial plateau. Scans of the 4, 14 and 66 % sites of the total length of the tibia were taken to represent predominantly trabecular bone at the distal metaphysis (4 %) and cortical bone at the diaphyseal site (14 and 66 %). The following parameters were analysed at the metaphyseal site: volumetric bone mineral density (mg/cm^3^), total bone mineral content (BMC, mg/mm) and total bone strength index, an estimate of axial compression strength, was calculated as the product of total bone cross-sectional area and the total density squared. At the diaphyseal sites, the following parameters were analysed: cortical volumetric bone mineral density, cortical BMC and stress–strain index of the polar axis (mm^3^), an estimate of diaphyseal torsional strength was determined and robustness was calculated as total cross-sectional area (mm^2^) divided by tibia length (mm) at the 4 and 14 % sites as described previously^(25–27)^. Image processing was performed using the Stratec software package (version 6.2).

### Statistical analyses

Data are reported as means and standard deviations. Baseline differences between females and males were determined using unpaired Student’s *t* tests. Two-way ANOVA was used to compare sex, intervention group and sex-by-intervention group interaction. Change scores were calculated by subtracting pre- from post-IMT values. Univariate and multiple linear regression models were used to examine the relationship between serum Mg concentrations and dietary Mg intakes or parameters of bone health. For dietary Mg intakes, univariate and multiple regression models adjusting for energy intake and accounting for age, race and BMI were used. For bone health, univariate and multiple regression models accounting for age, race, height, weight and intervention (model 1) and iCa (model 2) were used. Significance was demonstrated at *P* < 0·05. Data were analysed using SPSS version 24 (IBM Corp.).

## Results

### Participant characteristics pre-initial military training

Participant characteristics pre-IMT appear in Table 1. Participants were on average 19 years old, were predominantly White or Black and were of a healthy BMI (<25 kg/m^2^). Males were heavier and had less body fat than females (*P* < 0·001 for both). Dietary intake of Ca, vitamin D and Mg did not differ between females and males (*P* > 0·05 for all comparisons). Mean Ca-to-Mg intake ratios at baseline were high in both females and males compared with the optimal range of 1·7–2·6^(28)^. Approximately half of the participants met their respective EAR. Females had lower serum Mg concentrations pre-IMT compared with males (*P* = 0·001), and a higher percentage of females (35 %) were below the current serum Mg cut-off compared with males (18 %). Univariate and multiple regression models were used to determine whether dietary Mg was associated with serum Mg concentrations (online Supplementary Table 1). Unadjusted models and models adjusted for energy intake while accounting for age, race and BMI were used. Dietary Mg intake was not a significant predictor of serum Mg in males or females pre-IMT.

**Table 1.** Participant characteristics pre-military training* (Mean values and standard deviations; number and percentages; median values and interquartile range)

	Female		Male		*P*
	*n*	%	*n*	%	
Demographics					
Treatment					
Placebo	31	50	28	55	
Ca/vitamin D	31	50	23	45	
Age (years)					
Mean	19·1		18·7		0·119
sd	2·0		1·0		
Race					
White		69·4		84·3	
Black		21·0		15·7	
Asian		3·2		0	
Other		6·4		0	
BMI (kg/m^2^)					
Mean	23·4		24·4		0·058
sd	2·3		2·9		
Height (cm)					
Mean	162·9		176·1		<0·001
sd	5·4		5·7		
Weight (kg)					
Mean	62·3		75·8		<0·001
sd	6·9		10·6		
Body fat (%)					
Mean	22·0		12·0		<0·001
sd	4·3		4·9		
Dietary intake†					
Ca (mg/d)					
Median	1024·5		1047·7		0·192
IQR	695–1414		797–1572		
Vitamin D (μg /d)					
Median	5·7		6·2		0·066
IQR	2·7–8·0		4·2–9·6		
Mg (mg/d)					
Median	290·6		319·5		0·403
IQR	203–412		223–430		
Ca:Mg					
Median	3·6		3·7		0·387
IQR	2·8–4·3		3·0–4·5		
Serum Mg (mmol/l)					
Mean	0·79		0·85		0·001
sd	0·09		0·10		
<0·75 (mmol/l, %)	22	35	9	18	
0·75–0·95 (mmol/l, %)	39	63	37	73	
> 95 (mmol/l, %)	1	2	5	10	

EAR, estimated average requirement; IQR, interquartile range.

* Female, *n* 62; male, *n* 51.

† EAR (Mg): 300 mg/d (females 14–18 years), 340 mg/d (males 14–18 years), 255 mg/d (females 19–30 years), 330 mg/d (males 19–30 years).

### Relationship between serum magnesium and indices of bone health pre-initial military training

Univariate and multiple regression models that included serum Mg, age, race, height, weight (model 1) and iCa (model 2) for females and males pre-IMT appear in Table 2. Serum Mg concentrations were positively associated with BMC (*β* = 0·367, *P* = 0·004) and robustness (*β* = 0·393, *P* = 0·006) at the 4 % site, stress–strain index of the polaris axis (*β* = 0·334, *P* = 0·009) and robustness (*β* = 0·420, *P* = 0·004) at the 14 % site, and cortical BMC (*β* = 0·309, *P* = 0·009) and stress–strain index of the polaris axis (*β* = 0·314, *P* = 0·006) at the 66 % in females using model 2. In males, serum Mg was positively associated with osteocalcin (*β* = 0·327, *P* = 0·023) using model 2 and negatively associated with P1NP (R = –0·298, *P* = 0·034) and robustness at the 14 % site (*β* = –0·320, *P* = 0·047) using the univariate model; no significant relationships between serum Mg concentrations and bone parameters were observed in males in either of the multiple regression models.

**Table 2.** Relationship between serum magnesium concentration and bone parameters in females and males pre-military training

	Univariate		Model 1†			Model 2‡		
	*R*	*P*	*R*	*β*	*P*	*R*	*β*	*P*
Female								
PTH	0·053	0·685	0·353	0·110	0·435	0·446	0·064	0·640
OCN	–0·093	0·474	0·173	–0·091	0·536	0·173	–0·090	0·545
P1NP	–0·031	0·810	0·308	0·028	0·844	0·339	0·051	0·721
BAP	0·108	0·404	0·448*	–0·040	0·764	0·552*	–0·094	0·461
CTX	0·031	0·810	0·480*	0·084	0·524	0·509*	0·112	0·395
TRAP5b	–0·021	0·872	0·500*	–0·091	0·482	0·506*	–0·078	0·555
4 % BMC	0·247	0·072	0·656*	0·361	0·003	0·657*	0·367	0·004
4 % vBMD	–0·053	0·705	0·481*	–0·040	0·770	0·483	–0·034	0·810
4 % BSI	0·143	0·302	0·573*	0·231	0·075	0·575*	0·238	0·073
4 % robustness	0·298	0·029	0·507*	0·407	0·004	0·514*	0·393	0·006
14 % BMC	0·007	0·957	0·518*	0·064	0·634	0·518*	0·062	0·648
14 % vBMD	–0·095	0·493	0·345	–0·093	0·528	0·356	–0·107	0·476
14 % SSIp	0·247	0·072	0·635*	0·329	0·008	0·363*	0·334	0·009
14 % robustness	0·333	0·014	0·492*	0·428	0·003	0·494	0·420	0·004
66 % BMC	0·230	0·095	0·694*	0·319	0·006	0·696*	0·309	0·009
66 % vBMD	–0·103	0·459	0·249	–0·085	0·575	0·266	–0·100	0·515
66 % SSIp	0·217	0·116	0·719*	0·310	0·006	0·720*	0·314	0·006
Male								
PTH	0·068	0·635	0·407	0·110	0·452	0·417	0·108	0·467
OCN	0·322*	0·021	0·502*	0·329	0·021	0·517*	0·327	0·023
P1NP	–0·298*	0·034	0·551*	0·178*	0·184	0·642*	–0·179	0·159
BAP	0·177	0·215	0·304	0·163	0·285	0·314	0·147	0·345
CTX	0·008	0·955	0·364	0·027	0·856	0·407	0·036	0·812
TRAP5b	–0·015	0·616	0·492*	–0·007	0·958	0·524*	–0·031	0·821
4 % BMC	–0·139	0·399	0·620*	–0·086	0·567	0·651*	–0·085	0·576
4 % vBMD	0·043	0·797	0·419	0·054	0·757	0·540	0·059	0·725
4 % BSI	–0·037	0·825	0·459	–0·004	0·982	0·540	0·001	0·993
4 % robustness	–0·196	0·231	0·654*	–0·140	0·336	0·653*	–0·136	0·371
14 % BMC	–0·125	0·449	0·582*	–0·064	0·682	0·666*	–0·058	0·698
14 % vBMD	0·004	0·983	0·489	–0·087	0·601	0·561	–0·146	0·378
14 % SSIp	–0·287	0·077	0·773*	–0·225	0·071	0·787*	–0·234	0·065
14 % robustness	–0·320	0·047	0·718*	–0·267	0·051	0·719*	–0·272	0·056
66 % BMC	–0·173	0·293	0·628*	–0·194	0·198	0·628*	–0·173	0·268
66 % vBMD	–0·016	0·923	0·280	–0·101	0·583	0·460	–0·139	0·434
66 % SSIp	–0·294	0·069	0·708*	–0·246	0·075	0·716*	–0·231	0·103

PTH, parathyroid hormone; OCN, osteocalcin; P1NP, procollagen 1N-terminal peptide; BAP, bone alkaline phosphatase; CTX, c-telopeptide cross-links of type 1 collagen; TRAP5b, tartrate-resistant acid phosphatase 5b; BMC, bone mineral content; vBMD, volumetric bone mineral density; BSI, bone strength index; SSIp, stress–strain index of the polar axis; iCa, ionised Ca.

R for the model and standardised *β* coefficients and *P*-values for serum Mg are shown. PTH, *n* 51; bone parameters, *n* 39.

* *P* < 0·05 for the model.

† Covariates: serum Mg, age, race, height and weight.

‡ Covariates: serum Mg, iCa, age, race, height and weight.

The same univariate and multiple regression models that included serum Mg, age, race, height, weight (model 1) and iCa (model 2) were used to assess the relationship between dietary Mg intake and bone parameters in females and males pre-military training (online Supplementary Table 2). Dietary Mg was negatively associated with PTH in females for all models (univariate R = –0·373, *P* = 0·003; model 1 *β* = –0·327, *P* = 0·011; model 2 *β* = –0·304, *P* = 0·014), but not in males, was negatively associated with robustness at the 14 % site in males for most models (univariate R = –0·315, *P* = 0·051; model 1 *β* = –0·287, *P* = 0·031; model 2 *β* = –0·383, *P* = 0·010), but not in females, and was negatively associated with P1NP, a marker of bone formation (model 1 *β* = –0·273, *P* = 0·043), and 4 % robustness (univariate R = –0·319, *P* = 0·048) in males using one of the three models.

Females and males were separated based on the 0·75 mmol/l and 0·85 mmol/l serum Mg cut-offs pre-IMT, and markers of bone health were determined (online Supplementary Table 3). BMC at the 4 % site and robustness at the 4 and 14 % sites were greater (*P* < 0·05 for all) in females with a serum Mg concentration > 0·75 compared with ≤ 0·75 mmol/l pre-IMT. The observed differences in females pre-IMT were not observed when the serum Mg cut-off was increased to 0·85 mmol/l. There were no differences in any of the bone parameters in males pre-IMT regardless of the serum Mg cut-off.

### Change in body composition, dietary intake and biochemical measures during initial military training

Changes in body composition, dietary intake and biochemical measures from pre- to post-IMT appear in Table 3. Females lost more body fat than males (–5·02 ± 2·94 and −3·12 ± 3·63 kg, *P* = 0·002). Females and males in the Ca/vitamin D group consumed approximately more Ca and vitamin D during IMT than those in the placebo group. Dietary Mg did not change from pre- to post-IMT. Ca-to-Mg ratios increased in the Ca/vitamin D group (3·32 ± 1·85) compared with those in the placebo group (–0·16 ± 1·14; *P* < 0·001), and there was a greater increase in females (1·97 ± 2·34) compared with males (0·94 ± 2·17; *P* = 0·003).

**Table 3.** Change in body composition, dietary intake and biochemical measures pre- to post-military training* (Mean values and standard deviations)

	Female				Male						
	Placebo		Ca/vitamin D		Placebo		Ca/vitamin D		*P*		
	Mean	sd	Mean	sd	Mean	sd	Mean	sd	Sex	Group	Sex-by-group
Demographics											
BMI (kg/m^2^)											
Pre	24·1	2·1	22·7	2·3	24·6	3·0	24·1	2·9	–	–	–
Post	23·6	1·5	22·7	2·0	24·0	2·2	24·1	2·3	–	–	–
Δ	–0·54	1·24	–0·05	1·10	–0·56	1·32	–0·06	1·51	0·959	0·045	0·979
Weight (kg)											
Pre	65·2	6·3	59·4	6·3	74·5	10·1	77·5	11·3	–	–	–
Post	63·8	5·1	59·3	5·4	72·7	7·7	77·2	9·4	–	–	–
Δ	–1·42	3·40	–0·15	2·84	–1·74	3·99	–0·23	4·82	0·773	0·053	0·864
Body fat (%)											
Pre	23·6	4·3	20·3	3·7	11·5	3·9	12·6	6·0	–	–	–
Post	18·0	3·5	15·9	3·3	8·2	1·9	9·7	3·9	–	–	–
Δ	–5·61	3·08	–4·42	2·72	–3·36	3·59	–2·83	3·74	0·002	0·166	0·594
Dietary intake											
Ca (mg/d)											
Pre	985·5	492·5	1246·3	696·9	1278·1	678·3	1250·9	482·8	–	–	–
Post	1192·1	562·0	2198·3	662·9	973·6	428·6	2100·1	462·0	–	–	–
Δ	206·6	701·6	952·0	872·1	–304·5	737·2	849·2	624·5	0·032	<0·001	0·152
Vitamin D (μg/d)											
Pre	5·3	3·4	7·2	5·0	7·9	5·1	7·9	5·2	–	–	–
Post	7·1	5·2	34·8	5·5	5·4	3·5	32·6	3·8	–	–	–
Δ	1·8	6·1	27·6	6·2	–2·6	6·0	24·7	5·9	0·002	<0·001	0·535
Mg (mg/d)											
Pre	277·1	133·1	355·6	180·7	332·3	134·1	350·2	142·8	–	–	–
Post	333·1	114·5	351·2	125·6	327·3	109·5	360·6	107·5	–	–	–
Δ	56·0	166·0	–4·5	179·3	–5·0	149·0	10·3	150·8	0·456	0·466	0·222
Ca:Mg											
Pre	3·63	0·95	3·58	1·08	3·82	1·06	3·70	0·79	–	–	–
Post	3·90	1·07	7·24	1·88	3·17	0·61	6·57	1·24	–	–	–
Δ	0·27	0·85	3·66	2·12	–0·65	1·24	2·87	1·32	0·003	<0·001	0·810
Biochemical measures											
PTH (pg/ml)											
Pre	33·0	13·9	32·0	16·0	25·4	12·1	26·6	14·8	–	–	–
Post	33·3	12·8	35·8	16·0	23·4	10·9	21·9	13·1	–	–	–
Δ	0·33	15·80	3·75	20·94	–2·03	11·73	–4·69	12·28	0·077	0·900	0·317
iCa (nmol/l)											
Pre	1·20	0·03	1·20	0·03	1·22	0·04	1·21	0·04	–	–	–
Post	1·20	0·04	1·20	0·03	1·23	0·03	1·22	0·03	–	–	–
Δ	0·0006	0·04	0·002	0·03	0·006	0·04	0·011	0·03	0·347	0·671	0·773
25-(OH)D_3_ (ng/ml)											
Pre	31·2	11·0	24·0	7·5	31·7	9·6	27·0	8·0	–	–	–
Post	30·1	6·1	26·9	5·8	31·7	6·8	29·8	5·3	–	–	–
Δ	–1·42	7·54	1·78	5·48	–0·35	9·09	1·52	5·63	0·784	0·087	0·654
1,25-(OH)_2_D_3_ (ng/ml)											
Pre	84·6	24·7	77·9	32·8	82·9	18·6	79·4	22·2	–	–	–
Post	81·7	17·8	75·5	15·9	73·7	21·3	84·6	15·9	–	–	–
Δ	–2·29	26·48	–3·89	30·36	–9·23	24·74	5·19	23·43	0·835	0·213	0·120
Mg (mmol/l)											
Pre	0·76	0·08	0·81	0·09	0·82	0·10	0·89	0·08	–	–	–
Post	0·81	0·06	0·89	0·08	0·76	0·11	0·90	0·10	–	–	–
Δ	0·05	0·08	0·07	0·09	–0·05	0·11	0·01	0·08	<0·001	0·015	0·224
OCN (ng/ml)											
Pre	9·5	4·3	11·5	3·0	12·6	4·1	16·9	4·5	–	–	–
Post	8·0	2·9	9·0	3·0	10·8	3·7	12·4	4·1	–	–	–
Δ	–1·45	2·12	–2·48	3·26	–1·82	2·91	–4·49	3·42	0·035	0·001	0·143
P1NP (pg/ml)											
Pre	34·3	28·7	20·4	12·5	30·2	22·9	20·2	12·0	–	–	–
Post	32·5	21·0	19·7	10·8	33·5	31·6	22·1	9·6	–	–	–
Δ	–1·84	16·74	–0·78	7·11	3·22	17·46	1·95	12·88	0·149	0·968	0·663
BAP (U/l)											
Pre	26·2	7·6	32·1	8·6	34·2	11·1	40·4	13·1	–	–	–
Post	27·9	5·9	28·8	8·3	28·9	8·3	31·3	10·7	–	–	–
Δ	1·69	4·53	–3·21	7·84	–5·29	7·27	–9·15	7·51	<0·001	0·001	0·686
CTX (ng/ml)											
Pre	0·82	0·33	0·77	0·27	1·19	0·37	1·14	0·29	–	–	–
Post	0·74	0·23	0·63	0·22	0·85	0·31	0·82	0·33	–	–	–
Change	–0·08	0·27	–0·14	0·29	–0·34	0·35	–0·32	0·31	<0·001	0·701	0·489
TRAP5b (U/l)											
Pre	3·58	0·97	3·72	0·91	4·30	1·70	4·02	1·19	–	–	–
Post	4·08	0·96	3·26	0·91	4·22	1·37	3·41	0·77	–	–	–
Δ	0·50	0·81	–0·46	0·89	–0·07	0·87	–0·61	0·88	0·029	<0·001	0·203

PTH, parathyroid hormone; iCa, ionised Ca; 25-(OH)D_3_, 25-hydroxyvitamin D; 1,25-(OH)_2_D_3_, 1,25-dihydroxyvitamin D; OCN, osteocalcin; P1NP, procollagen 1N-terminal peptide; BAP, bone alkaline phosphatase; CTX, c-telopeptide cross-links of type 1 collagen; TRAP5b, tartrate-resistant acid phosphatase 5b.

* Female placebo, *n* 31; female Ca/vitamin D, *n* 31; male placebo, *n* 28; male Ca/vitamin D, *n* 23.

Sex and group differences were observed for the change in serum Mg, osteocalcin, BAP and TRAP5b with training. Serum Mg increased during IMT in females compared with males (0·06 ± 0·08 and −0·02 ± 0·10 mmol/l, *P* < 0·001). Osteocalcin (–1·97 ± 2·78 and −3·03 ± 3·39 ng/l), BAP (–0·76 ± 2·78 and −7·03 ± 7·56 U/l), CTX (–0·11 ± 0·28 and −0·33 ± 0·33 ng/ml) and TRAP5b (–0·02 ± 0·97 and −0·31 ± 0·91 U/l) declined in females and males during IMT, with a greater decline in males compared with females (*P* < 0·05 for all comparisons).

Serum Mg increased during IMT in those consuming Ca/vitamin D compared with placebo (0·046 ± 0·09 and 0·001 ± 0·11 mmol/l, *P* = 0·015). Osteocalcin (–3·34 ± 3·45 and −1·63 ± 2·51 ng/l), BAP (–5·74 ± 8·18 and −1·62 ± 6·90 U/l) and TRAP5b (–0·52 ± 0·88 and 0·23 ± 0·88 U/l) decreased in those consuming Ca/vitamin D compared with placebo (*P* < 0·05 for all comparisons).

### Relationship between change in serum magnesium and change in indices of bone turnover during initial military training

Univariate and multiple regression models were used to determine if changes in serum Mg during IMT were associated with changes in bone turnover markers (online Supplementary Table 4). No significant relationships were observed in females. In males, change in serum Mg was positively associated with change in TRAP5b using model 1 (*β* = 0·313, *P* = 0·021) and model 2 (*β* = 0·333, *P* = 0·014).

## Discussion

The major findings from the current study were (1) dietary Mg intakes were below the EAR and hypomagnesemia was observed in a large proportion of females and males prior to and during IMT, (2) serum Mg was associated with parameters of bone health in females, but not in males and (3) Ca/vitamin D supplementation during IMT increased serum Mg compared with placebo. These findings indicate that Mg may be a nutrient of concern for military recruits, which may be particularly relevant given the increased incidence of tibial stress fractures during military training, as compared with other times in a service member’s career.

Median dietary Mg intakes were below recommendations pre- (Table 1) and post-IMT (data not shown), respectively. The finding that approximately half of males and females did not meet the EAR for Mg pre- or post-IMT is consistent with the NHANES 2007–2010 data used by the 2015 Dietary Guidelines Advisory Committee^(3)^. However, the current EAR was based on a study in 16 men and 18 women who consumed a self-selected diet^(29)^. Since that time, Mg balance data from twenty-seven studies were compiled and an EAR of 165 mg/d (RDA of 250 mg/d), regardless of sex and age, was suggested^(30)^. Using these criteria, 16 % and 7 % of females and 8 % and 4 % of males were below the EAR pre- and post-IMT, respectively. However, it should be noted that the values above are for approximately 70 kg individuals, vary with body weight, and do not consider the physical demands of military training. Regardless, choosing foods rich in Mg, including whole grains, nuts and seeds, and vegetables, remains an important consideration.

The current lower cut-off for serum Mg (0·75 mmol/l) was developed based on serum Mg concentrations from a representative national sample of the US population between 1971 and 1974^(4)^. Based on these criteria, females were twice as likely (35 %) to be below the cut-off pre-IMT compared with males (18 %) (Table 1). Increased risk for conditions associated with low Mg intake or hypomagnesemia, such as hypertension, CVD, type 2 diabetes and osteoporosis, have been observed with serum Mg concentrations within the current reference interval. Thus, current evidence has led some to suggest that the lower cut-off could be increased to 0·85 mmol/l^(5)^. If the lower cut-off were to increase to 0·85 mmol/l, the percentage of females (81 %) and males (49 %) below the cut-off in the current study would more than double. Interestingly, BMC at the 4 % site and total and cortical cross-sectional area at the 4 % and 14 % sites were elevated in females with serum Mg > 0·75 mmol/l compared with ≤ 0·75 mmol/l, but not when the cut-off was increased to 0·85 mmol/l. Although these findings are not causative, serum Mg was a significant predictor of each of these variables in multiple regression models, suggesting that a lower cut-off of 0·75 mmol/l may be associated with lower bone mineral status.

Exercise is known to redistribute Mg in the body^(16)^. In general, short-term, high-intensity exercise results in a transient increase of about 5–15 %, while prolonged endurance exercise reduces circulating concentrations of Mg (reviewed in^(16,31)^). In the current study, the serum Mg response to military training varied based on sex. Serum Mg increased with military training in females, but not in males. The source of the increase in serum Mg in females is unclear; however, exercise-induced increases in serum Mg concentration may reflect Mg being pulled from the bone surface to help maintain serum Mg concentrations^(2)^. Rude and colleagues performed a series of studies that induced Mg deficiency by feeding rats 0·4 %, 10 %, 25 % and 50 % of the Mg requirement^(9–13)^. Female rats fed even the most modest restriction (50 % of the Mg requirement) had reduced bone Mg concentrations, trabecular bone volume and trabecular BMC at the distal femur compared with controls^(9)^. Importantly, the detrimental effects of Mg restriction on bone health were observed in the absence of a decline in serum Mg concentrations^(9)^. In the current study, females had a higher prevalence of hypomagnesemia compared with males and serum Mg in females was positively associated with bone parameters pre-IMT. Despite these findings, the increase in serum Mg in females during IMT could not be explained by an increase in bone turnover markers. Alternative explanations for the increase in serum Mg post-IMT include shifts in plasma volume^(32,33)^, increased muscle breakdown and release of Mg into circulation, as approximately one-third of total body Mg is found in skeletal muscle^(1)^, and/or reduced Mg utilisation in females compared with males.

Serum Mg increased during IMT in those consuming Ca/vitamin D compared with placebo, but like the increase in serum Mg that occurred in females during IMT, could not be explained by an increase in bone turnover markers. Previous studies have demonstrated direct effects of vitamin D on intestinal Mg absorption^(18)^ and/or the modulation of PTH by Mg, and an indirect increase in Mg absorption through 1,25-dihydroxyvitamin D synthesis. Either mechanism would be consistent with *in vitro* and human data that demonstrate that reductions in Mg enhance PTH secretion, whereas increased Mg inhibits PTH secretion^(34,35)^. Rats consuming a diet containing 50 % of the Mg requirement had reduced serum Ca and 1,25-dihydroxyvitamin D and increased PTH concentrations and osteoclast numbers^(9)^. In the current study, females had lower serum Mg and increased PTH concentrations compared with males pre-IMT; however, it should be noted that serum Mg was not associated with PTH in the current study.

This study has several limitations. First, it was a secondary analysis of a randomised, double-blind, placebo-controlled trial examining the effects of Ca/vitamin D supplementation on bone health in military recruits and was therefore not designed to specifically look at the outcomes reported. Secondly, serum Mg as a measure of total body Mg status has known limitations^(1)^. And lastly, the numerous study outcomes increase the risk of making a type I error. Strengths of the current study include the ability to relate serum Mg concentrations to indicators of bone health using both imaging (peripheral quantitative computed tomography) and biochemical markers in apparently healthy females and males.

In conclusion, serum Mg was positively associated with some peripheral quantitative computed tomography indices of bone health in healthy young females prior to military training. Serum Mg increased during military training in females, but not in males. These findings indicate that Mg may be a nutrient of concern for female military recruits as Mg may be mobilised from bone to increase circulating concentrations during training. Further research is required to determine dietary Mg requirements during military or athletic training.

## Supplementary material

For supplementary material accompanying this paper visit https://doi.org/10.1017/S0007114521004669. click here to view supplementary material
