# Supplementary material for: Divergent effects of sex and calcium/vitamin D supplementation on serum magnesium and markers of bone structure and function during initial military training
Source: Br J Nutr. 2021 Nov 24;128(9):1730–7. doi: 10.1017/S0007114521004669 (PMC9592948; doi:10.1017/S0007114521004669)
Supplement: Supplementary file 1 [file S0007114521004669sup001.docx]

**Supplementary Table 1.** Relationship between dietary magnesium intake and serum magnesium concentrations in females and males pre- military training

|  | Univariate^1^ | | Univariate^2^ | | Model 1^3^ | | | Model 2^4^ | | |
| --- | --- | --- | --- | --- | --- | --- | --- | --- | --- | --- |
|  | R | P | R | P | R | β | P | R | β | P |
| Female | 0.168 | 0.191 | 0.123 | 0.342 | 0.471* | 0.446 | 0.119 | 0.456* | 0.173 | 0.182 |
| Male | 0.089 | 0.536 | -0.043 | 0.764 | 0.312 | 0.010 | 0.973 | 0.315 | -0.067 | 0.636 |

^1^Variables: serum magnesium and dietary magnesium.

^2^Variables: serum magnesium and dietary magnesium/kcals.

^3^Covariates: dietary magnesium, age, race, BMI.

^4^Covariates: dietary magnesium/kcals, age, race, BMI.

Univariate regressions for dietary magnesium and dietary magnesium adjusted for energy (kcals); R for the model and standardized β coefficients and P-values for dietary magnesium are shown. Female, n= 62; male, n=51. *P<0.05 for the model.

**Supplementary Table 2.** Relationship between dietary magnesium and bone parameters in females and males pre-military training

|  | Univariate | | Model 1^1^ | | | Model 2^2^ | | |
| --- | --- | --- | --- | --- | --- | --- | --- | --- |
|  | R | P | R | β | P | R | β | P |
| Female |  |  |  |  |  |  |  |  |
| PTH | -0.373* | **0.003** | 0.463* | -0.327 | **0.011** | 0.531* | -0.304 | **0.014** |
| OCN | -0.049 | 0.707 | 0.154 | -0.021 | 0.877 | 0.155 | -0.023 | 0.871 |
| P1NP | -0.193 | 0.132 | 0.353 | -0.179 | 0.176 | 0.384 | -0.192 | 0.146 |
| BAP | -0.061 | 0.638 | 0.467* | -0.143 | 0.252 | 0.557* | -0.115 | 0.330 |
| CTX | -0.132 | 0.307 | 0.503* | -0.174 | 0.156 | 0.419 | -0.189 | 0.120 |
| TRAP5b | -0.009 | 0.943 | 0.499* | -0.078 | 0.524 | 0.508* | -0.087 | 0.480 |
| 4% BMC | 0.099 | 0.478 | 0.591* | 0.192 | 0.123 | 0.591* | 0.193 | 0.127 |
| 4% vBMD | 0.098 | 0.480 | 0.509* | 0.175 | 0.187 | 0.510* | 0.175 | 0.192 |
| 4% BSI | 0.122 | 0.381 | 0.574* | 0.230 | 0.072 | 0.574* | 0.230 | 0.075 |
| 4% robustness | 0.107 | 0.440 | 0.680* | 0.113 | 0.433 | 0.377 | 0.114 | 0.426 |
| 14% BMC | -0.101 | 0.468 | 0.515* | 0.019 | 0.886 | 0.515* | 0.019 | 0.886 |
| 14% vBMD | -0.122 | 0.380 | 0.346 | -0.096 | 0.504 | 0.353 | -0.095 | 0.511 |
| 14% SSIp | -0.079 | 0.569 | 0.556* | 0.038 | 0.763 | 0.556* | -0.285 | 0.034 |
| 14% robustness | 0.132 | 0.342 | 0.336 | 0.189 | 0.193 | 0.353 | 0.190 | 0.192 |
| 66% BMC | 0.008 | 0.952 | 0.633* | 0.105 | 0.376 | 0.642* | 0.106 | 0.372 |
| 66% vBMD | 0.100 | 0.474 | 0.264 | 0.124 | 0.401 | 0.277 | 0.125 | 0.401 |
| 66% SSIp | -0.050 | 0.722 | 0.660* | 0.068 | 0.554 | 0.661* | 0.068 | 0.557 |
| Male |  |  |  |  |  |  |  |  |
| PTH | 0.083 | 0.564 | 0.395 | -0.027 | 0.857 | 0.405 | 0.017 | 0.920 |
| OCN | 0.156 | 0.275 | 0.413 | 0.131 | 0.377 | 0.422 | 0.084 | 0.609 |
| P1NP | -0.215 | 0.130 | 0.582* | -0.273 | **0.043** | 0.634* | -0.156 | 0.270 |
| BAP | 0.178 | 0.211 | 0.303 | 0.164 | 0.292 | 0.317 | 0.173 | 0.318 |
| CTX | 0.186 | 0.191 | 0.400 | 0.181 | 0.227 | 0.532* | 0.121 | 0.463 |
| TRAP5b | 0.202 | 0.155 | 0.507* | 0.129 | 0.359 | 0.527* | 0.077 | 0.616 |
| 4% BMC | -0.052 | 0.752 | 0.617* | -0.051 | 0.728 | 0.662* | -0.170 | 0.292 |
| 4% vBMD | 0.233 | 0.154 | 0.444 | 0.166 | 0.323 | 0.538 | 0.034 | 0.850 |
| 4% BSI | 0.094 | 0.568 | 0.461 | 0.054 | 0.743 | 0.544 | -0.078 | 0.665 |
| 4% robustness | -0.319 | **0.048** | 0.350 | -0.240 | 0.085 | 0.681* | -0.270 | 0.091 |
| 14% BMC | 0.126 | 0.446 | 0.587* | 0.099 | 0.515 | 0.665* | -0.042 | 0.793 |
| 14% vBMD | 0.107 | 0.518 | 0.484 | 0.044 | 0.789 | 0.555 | -0.122 | 0.494 |
| 14% SSIp | -0.150 | 0.362 | 0.761* | -0.167 | 0.174 | 0.795* | 0.039 | 0.764 |
| 14% robustness | -0.315 | 0.051 | 0.727* | -0.287 | **0.031** | 0.749* | -0.383 | **0.010** |
| 66% BMC | -0.021 | 0.900 | 0.607* | -0.083 | 0.574 | 0.618* | -0.131 | 0.436 |
| 66% vBMD | 0.008 | 0.959 | 0.266 | -0.033 | 0.853 | 0.491 | -0.251 | 0.184 |
| 66% SSIp | -0.130 | 0.431 | 0.696* | -0.197 | 0.148 | 0.728* | -0.293 | 0.052 |

^1^Covariates: serum magnesium, age, race, height, and weight.

^2^Covariates: serum magnesium, iCa, age, race, height, and weight.

R for the model and standardized β coefficients and P-values for dietary magnesium are shown. PTH, n=51; bone parameters, n=39. *P<0.05 for the model. BAP, bone alkaline phosphatase; BMC, bone mineral content; BSI, bone strength index; BSI, bone strength index; CTX, c-telopeptide cross-links of type 1 collagen; OCN, osteocalcin; P1NP, procollagen 1N-terminal peptide; PTH, parathyroid hormone; SSIp, stress strain index of the polar axis; TRAP5b, tartrate-resistant acid phosphatase 5b; vBMD, volumetric bone mineral density.

**Supplementary Table 3.** Serum magnesium concentrations and bone parameters separated based on serum magnesium cutoffs in females and males pre- military training^1^

|  | Serum magnesium, *mmol/L* | | | |  | Serum magnesium, *mmol/L* | | | |
| --- | --- | --- | --- | --- | --- | --- | --- | --- | --- |
|  | n | ≤ 0.75 | n | > 0.75 |  | n | ≤ 0.85 | n | > 0.85 |
| Female |  |  |  |  |  |  |  |  |  |
| Serum magnesium, *mmol/L* | 22 | 0.69 ± 0.04 | 40 | 0.84 ± 0.06* |  | 50 | 0.76 ± 0.07 | 12 | 0.91 ± 0.04* |
| Dietary magnesium, *mg/d* | 22 | 282.5 ± 145.9 | 40 | 335.0 ± 169.5 |  | 50 | 308.4 ± 161.9 | 12 | 349.7 ± 166.6 |
| PTH, *pg/mL* | 22 | 32.8 ± 14.8 | 40 | 32.3 ± 15.1 |  | 35 | 30.9 ± 13.3 | 27 | 34.7 ± 20.5 |
| 4% BMC, *mg/mm* | 19 | 291.9 ± 34.2 | 35 | 318.0 ± 41.4* |  | 33 | 309.2 ± 43.6 | 21 | 308.3 ± 20.6 |
| 4% vBMD, *mm/cm^3^* | 19 | 326.9 ± 24.2 | 35 | 328.1 ± 38.4 |  | 33 | 332.7 ± 32.6 | 21 | 319.7 ± 38.2 |
| 4% BSI, *mm/mm^4^* | 19 | 95.8 ± 16.4 | 35 | 104.8 ± 20.9 |  | 33 | 103.3 ± 20.4 | 21 | 99.1 ± 15.3 |
| 4% robustness, *mm^2^/mm* | 19 | 2.4 ± 0.2 | 35 | 2.6 ± 0.3* |  | 33 | 2.5 ± 0.3 | 21 | 2.6 ± 0.3 |
| 14% BMC, *mg/mm* | 19 | 185.3 ± 22.2 | 35 | 187.8 ± 27.7 |  | 33 | 191.4 ± 26.7 | 21 | 179.9 ± 18.9 |
| 14% vBMD, *mg/cm^3^* | 19 | 1136.0 ± 24.1 | 35 | 1133.2 ± 21.2 |  | 33 | 1134.8 ± 23.3 | 21 | 1133.1 ± 15.6 |
| 14% SSIp | 19 | 1260.2 ± 225.4 | 35 | 1393.3 ± 262.2 |  | 33 | 1345.7 ± 265.0 | 21 | 1347.7 ± 218.3 |
| 14% robustness, *mm^2^/mm* | 19 | 1.0 ± 0.1 | 35 | 1.2 ± 0.2* |  | 33 | 1.1 ± 0.2 | 21 | 1.2 ± 0.1 |
| 66% BMC, *mg/mm* | 19 | 311.3 ± 36.1 | 35 | 322.7 ± 39.7 |  | 33 | 317.5 ± 41.0 | 21 | 324.5 ± 23.2 |
| 66% vBMD, *mg/cm^3^* | 19 | 1130.3 ± 26.7 | 35 | 1128.4 ± 17.4 |  | 33 | 1130.0 ± 21.3 | 21 | 1124.3 ± 19.1 |
| 66% SSIp | 19 | 2160.6 ± 384.0 | 35 | 2305.7 ± 419.7 |  | 33 | 2253.4 ± 440.8 | 21 | 2260.8 ± 210.0 |
| Male |  |  |  |  |  |  |  |  |  |
| Serum magnesium, *mmol/L* | 9 | 0.69 ± 0.02 | 42 | 0.88 ± 0.07* |  | 25 | 0.77 ± 0.06 | 26 | 0.92 ± 0.06* |
| Dietary magnesium, *mg/d* | 9 | 364.6 ± 150.0 | 42 | 335.2 ± 135.4 |  | 25 | 329.8 ± 118.4 | 26 | 350.6 ± 154.4 |
| PTH, *pg/mL* | 9 | 23.3 ± 14.4 | 42 | 26.5 ± 13.2 |  | 33 | 26.0 ± 12.0 | 18 | 25.9 ± 14.7 |
| 4% BMC, *mg/mm* | 6 | 416.6 ± 60.2 | 33 | 413.3 ± 63.2 |  | 25 | 412.0 ± 58.8 | 14 | 417.0 ± 66.0 |
| 4% vBMD, *mm/cm^3^* | 6 | 337.5 ± 27.4 | 33 | 356.0 ± 41.6 |  | 25 | 352.7 ± 27.5 | 14 | 354.1 ± 48.9 |
| 4% BSI, *mm/mm^4^* | 6 | 141.3 ± 27.3 | 33 | 148.6 ± 34.4 |  | 25 | 145.7 ± 26.9 | 14 | 150.6 ± 38.4 |
| 4% robustness, *mm^2^/mm* | 6 | 3.1 ± 0.3 | 33 | 2.8 ± 0.3 |  | 25 | 2.9 ± 0.3 | 14 | 2.9 ± 0.4 |
| 14% BMC, *mg/mm* | 6 | 234.7 ± 30.6 | 33 | 239.5 ± 36.3 |  | 25 | 237.8 ± 32.9 | 14 | 239.6 ± 37.9 |
| 14% vBMD, *mg/cm^3^* | 6 | 1116.1 ± 13.4 | 33 | 1114.9 ± 21.7 |  | 25 | 1115.0 ± 22.0 | 14 | 1115.3 ± 19.3 |
| 14% SSIp | 6 | 1927.0 ± 366.6 | 33 | 1848.5 ± 398.0 |  | 25 | 1854.3 ± 345.2 | 14 | 1871.8 ± 427.7 |
| 14% robustness, *mm^2^/mm* | 6 | 1.3 ± 0.2 | 33 | 1.3 ± 0.2 |  | 25 | 1.3 ± 0.1 | 14 | 1.3 ± 0.2 |
| 66% BMC, *mg/mm* | 6 | 393.3 ± 67.6 | 33 | 392.9 ± 48.4 |  | 25 | 393.1 ± 52.9 | 14 | 392.8 ± 50.1 |
| 66% vBMD, *mg/cm^3^* | 6 | 1099.4 ± 10.8 | 33 | 1098.7 ± 22.0 |  | 25 | 1094.5 ± 22.0 | 14 | 1102.5 ± 18.9 |
| 66% SSIp | 6 | 3307.0 ± 643.8 | 33 | 3204.7 ± 627.2 |  | 25 | 3341.3 ± 654.6 | 14 | 3116.8 ± 589.2 |

^1^Values are means ± SD. *P<0.05 compared to respective cutoff. BMC, bone mineral content; BSI, bone strength index; PTH, parathyroid hormone; SSIp, stress strain index of the polar axis; vBMD, volumetric bone mineral density.

**Supplementary Table 4.** Relationship between change in serum magnesium and change in bone parameters in females and males pre- to post- military training

|  | Univariate | | Model 1^1^ | | | Model 2^2^ | | |
| --- | --- | --- | --- | --- | --- | --- | --- | --- |
|  | R | P | R | β | P | R | β | P |
| Female |  |  |  |  |  |  |  |  |
| Δ PTH | -0.051 | 0.692 | 0.290 | -0.002 | 0.987 | 0.461 | 0.029 | 0.830 |
| Δ OCN | -0.057 | 0.659 | 0.317 | -0.040 | 0.774 | 0.338 | 0.001 | 0.993 |
| Δ P1NP | 0.030 | 0.818 | 0.234 | 0.060 | 0.672 | 0.268 | 0.087 | 0.546 |
| Δ BAP | 0.051 | 0.692 | 0.564 | -0.055 | 0.717 | 0.624* | 0.126 | 0.283 |
| Δ CTX | 0.095 | 0.461 | 0.408 | 0.131 | 0.326 | 0.462 | 0.140 | 0.293 |
| Δ TRAP5b | 0.064 | 0.619 | 0.621* | 0.080 | 0.485 | 0.673* | 0.127 | 0.256 |
| Male |  |  |  |  |  |  |  |  |
| Δ PTH | 0.177 | 0.213 | 0.408 | 0.212 | 0.152 | 0.412 | 0.232 | 0.134 |
| Δ OCN | -0.275 | 0.051 | 0.468 | -0.184 | 0.199 | 0.560* | -0.164 | 0.240 |
| Δ P1NP | 0.029 | 0.838 | 0.328 | 0.019 | 0.903 | 0.315 | 0.013 | 0.933 |
| Δ BAP | -0.116 | 0.416 | 0.331 | 0.086 | 0.476 | 0.484 | -0.027 | 0.855 |
| Δ CTX | 0.057 | 0.690 | 0.489* | 0.037 | 0.789 | 0.500 | 0.057 | 0.697 |
| Δ TRAP5b | 0.200 | 0.159 | 0.571* | 0.313 | **0.021** | 0.622* | 0.333 | **0.014** |

^1^Covariates: Δ serum magnesium, age, race, height, weight, and intervention group.

^2^Covariates: Δ serum magnesium, Δ iCa, age, race, height, weight, and intervention group.

R for the model and standardized β coefficients and P-values for change in serum magnesium are shown. PTH, n=51; bone parameters, n=39. *P<0.05 for the model. BAP, bone alkaline phosphatase; CTX, c-telopeptide cross-links of type 1 collagen; OCN, osteocalcin; P1NP, procollagen 1N-terminal peptide; PTH, parathyroid hormone; TRAP5b, tartrate-resistant acid phosphatase 5b.
